# How to Read a Next-Generation Sequencing Report for AML and MDS? What Hematologists Need to Know

**DOI:** 10.3390/jcm14248681

**Published:** 2025-12-08

**Authors:** Salvatore Perrone, Cristina Tresoldi, Silvia Rigamonti, Matteo Molica, Nadezda Zhdanovskaya, Laura Cicconi

**Affiliations:** 1 Department of Hematology, Santa Maria Goretti Hospital, Polo Universitario Pontino, 04100 Latina, Italy; sperrone@hotmail.it (S.P.); l.cicconi@ausl.latina.it (L.C.); 2 Molecular Hematology Unit, IRCCS San Raffaele Scientific Institute, Via Olgettina, 60, 20132 Milan, Italy; rigamonti.silvia@hsr.it; 3 Department of Hematology-Oncology, Azienda Ospedaliera Pugliese-Ciaccio, 88100 Catanzaro, Italy; matteomol@hotmail.it; 4 Hematology, Department of Translational and Precision Medicine, Sapienza University, 00161 Rome, Italy; nadezda.zhdanovskaya@uniroma1.it

**Keywords:** acute myeloid leukemia (AML), next-generation sequencing (NGS), NGS-report, myelodysplastic neoplasms (MDS), measurable residual disease (MRD), germline predisposition to myeloid neoplasms

## Abstract

Acute myeloid leukemia (AML) and myelodysplastic neoplasms (MDS) are clonal hematopoietic malignancies in which next-generation sequencing (NGS) has become integral for diagnosis, classification, risk stratification, and measurable residual disease (MRD) monitoring. Traditional cytogenetic and PCR-based assays remain useful, but targeted NGS panels now represent the standard of care, providing rapid and sensitive detection of recurrent gene mutations, structural variants, and gene fusions. Whole-genome, whole-exome, and RNA sequencing and long-read platforms expand the spectrum of detectable alterations, though targeted panels remain most practical for routine diagnostics. Bioinformatic pipelines and quality metrics—including read length, sequencing depth, and coverage—are critical for accurate variant calling, with validation often required for variants of uncertain significance or those near detection thresholds. NGS is now embedded in diagnostic frameworks, including the WHO 2022 and ICC classifications, which incorporate recurrently mutated genes such as *TP53*, *ASXL1*, *RUNX1*, and *FLT3*. These data inform prognostic models, with ELN-2022 defining adverse-risk AML subgroups for patients treated with intensive chemotherapy, ELN-2024 AML for patients treated with less-intensive therapies, and the IPSS-M refining MDS risk categories by integrating mutational data. NGS also enables MRD monitoring, with gene panels and PCR-NGS hybrid approaches (e.g., for *FLT3*-ITD) showing increasing clinical utility, though standardization is still lacking. Furthermore, diagnostic NGS frequently uncovers germline predisposition syndromes (e.g., *DDX41*, *GATA2*), with significant implications for treatment decisions and donor selection in transplantation. In this manuscript, we review the advantages, limitations, and future perspectives of NGS in the clinical management of AML and MDS with a particular emphasis on the biological and technical principles underlying its use in these diseases. Furthermore, we discuss how NGS findings may influence diagnosis, prognostic classification, and therapeutic decision-making within current clinical frameworks. Our aim is to provide a comprehensive overview of NGS fundamentals to support clinicians in navigating the increasing complexity of molecular data in daily practice.

## 1. Introduction

Acute myeloid leukemia (AML) and myelodysplastic neoplasms (abbreviated MDS) are clonal hematopoietic neoplasms, characterized by at least one somatic mutation in 96% of cases [1]. Although the WHO fifth edition still allows for the diagnosis of AML on the basis of differentiation in cases lacking defining genetic abnormalities, and dysplasia in MDS may still correspond to morphological criteria [2], in both diseases diagnosis increasingly relies on extensive laboratory workups aimed at identifying specific genetic features. In this contest, the classical PCR-based and cytogenetic techniques have been rapidly complemented by next-generation sequencing (NGS). It is now standard of care to perform NGS-based mutational profiling in all patients with AML, with direct implications for treatment selection and prognostication [3,4]. Indeed, NGS enables the simultaneous sequencing of millions of DNA fragments, providing comprehensive insights into the genome structure, genetic variations, gene expression profiles, and epigenetic alterations of neoplastic cells [5]. However, the broad availability and reduced cost of NGS in AML/MDS has also increased the uncertainty for both hematologists and patients who must interpret the expanding volume of molecular information from sequencing reports. Therefore, we conducted this review to support clinicians, similarly to previously published guidance for oncologists like ESMO recommendations, on the use of NGS in advanced cancers [6,7].

## 2. NGS Techniques

NGS is based on the principle of massively parallel sequencing, in which millions of DNA fragments are sequenced simultaneously [8]. Different NGS approaches can be applied depending on clinical and research needs, allowing the sequencing of the whole genome, exome, or transcriptome [9]. Whole-genome sequencing (WGS) provides a complete picture of genetic variations; whole-exome sequencing (WES) focuses on protein-coding regions; RNA sequencing can be useful for identification of fusion genes; whereas targeted sequencing panels, the most widely used in clinical practice, concentrate on sets of genes recurrently mutated in MDS and AML [10].

### 2.1. NGS Workflow

The typical NGS workflow involves extraction of DNA or RNA from the sample, library preparation through fragmentation and adaptor ligation (as in hybridization capture-based assays) or target enrichment in amplification-based assays, followed by sequencing [9].

In hybridization capture, DNA is randomly fragmented and sequencing adapters are ligated to the ends. Biotinylated probes are then used to selectively isolate the regions of interest.

In contrast, amplicon sequencing relies on primers specifically designed to amplify specific targets by PCR, after which adapters are added to prepare the amplified products for sequencing.

The choice between these two approaches depends on several factors such as number of targets, workflow complexity, and cost per sample.

In both methods, patient-specific index sequences are incorporated in addition to adapters required for sequencing on the instrument. These indexes allow multiple libraries to be pooled in the same sequencing run, enabling simultaneous analysis of multiple samples, even from different patients [11].

Different platforms rely on distinct sequencing techniques, including sequencing by synthesis, sequencing by hybridization, sequencing by ligation, and electric impedance-based sequence detection [5]. The most widely used in clinical practice is sequencing by synthesis (SBS), employed by Illumina instruments, in which fluorescently labeled nucleotides are incorporated and detected in real time. A semiconductor-based variation in SBS, used by Ion Torrent systems, detects the release of H^+^ ions instead of fluorescence following nucleotide incorporation [12]. Both approaches require prior amplification of DNA fragments: Illumina platforms use bridge amplification in which fragments are loaded onto a flow cell where they fold over to form bridges, whereas Ion Torrent amplifies fragments after binding them individually to magnetic beads [13].

More recently, long-read sequencing technologies have emerged. Single-molecule real-time sequencing (SMRT) by Pacific Biosciences (Menlo Park, CA, USA) produces highly accurate “HiFi” long reads, while nanopore sequencing (Oxford Nanopore Technologies, Oxford, UK) measures changes in ionic current as nucleic acids pass through protein pores, enabling ultra-long reads and real-time analysis [12]. While short-read SBS remains the gold standard for routine AML and MDS diagnostics, long-read platforms are increasingly being explored for the detection of structural variants, fusion genes, and transcript isoforms [9,14]. In these cases, amplification is not required, as nucleic acid fragments can be sequenced directly [11].

Bioinformatic pipelines then align reads to a reference genome, call and annotate variants, and generate interpretable data. Key biological metrics following alignment to a reference genome—excluding run quality check—include read length, sequencing depth and coverage, duplication rate, limit of detection (LoD), CG-bias, as CG-rich fragments may show a lower coverage, and some other assay-specific parameters that will be shortly described below [9,11,12]. In the clinical setting, results often require validation, and variant interpretation must integrate allele frequency, functional annotation, and clinical relevance [6,9].

In NGS, a read represents the nucleotide sequence obtained from a single DNA or RNA fragment during sequencing. Read length varies by platform, but in clinical oncology and hematology, most targeted DNA and RNA panels produce reads of 50–300 bp (commonly 100–250 bp), which are sufficient to detect point mutations, small indels, and gene fusions. However, short reads can limit the precise detection of long insertions such as some *FLT3* ITD variants or long deletions like *CALR* type 1 mutations [5,15,16,17]. Whole-genome sequencing and long-read technologies can generate longer reads, typically in the range of 10–100 kilobases and sometimes several megabases, enabling the detection of structural variants, complex rearrangements, and full-length transcript isoforms [18,19,20]. Technically, reads may also fail to map uniquely, remain unmapped or, in RNA-seq, map to uninformative transcripts [21].

NGS platforms include built-in measures of read quality, ensuring high accuracy for variant detection [22].

The sequencing coverage indicates the proportion of a reference region (genome, exome, transcriptome, or targeted gene panel) that has been sequenced at least once. Insufficient coverage can lead to errors or uncertainty, making it difficult to distinguish between a true genetic finding and a technical artifact, and complicate downstream bioinformatical analysis [14]. Some fragments like CG-rich regions, polyploid genes, or very rare variants may require increased sequencing depth or coverage [21].

Sequencing depth refers to the average number of times each nucleotide in a DNA or RNA sample is read during an NGS run, usually expressed as a multiple (e.g., 100×). Higher depth increases confidence in variant detection [21]. For RNA-seq and targeted RNA sequencing, reporting the total number of reads per sample is essential, as it strongly impacts sensitivity for gene expression analysis, detection of fusion transcripts, and identification of low-abundance isoforms [23]. In human whole-transcriptome (mRNA) RNA-seq, 20–30 million reads per sample are generally sufficient, with 30–50 million reads recommended for higher sensitivity. Detection of low-expression transcripts or detailed splicing may require significantly deeper sequencing, while targeted RNA panels may need as few as 1–3 million reads per sample due to their focused design [23,24,25,26].

Different NGS types achieve different sequencing depths. Generally, WGS provides the sequencing depth that ranges at 30–60× and the exome-only coverage with WES reaches the depth range around 100–200× which may be insufficient for detecting low-frequency variants. In contrast, targeted gene panels focus on a smaller genomic region and achieve a higher depth of sequencing (typically at 500–1000×) improving sensitivity for rare variants [27].

The sequencing depth required for reliable detection of low-frequency variants remains a critical issue in NGS diagnostics. The guidelines of the Association for Molecular Pathology and College of American Pathologists (AMP/CAP) recommend a minimum coverage of >250× per amplicon for routine somatic variant detection in targeted oncology panels typically enabling detection of variants with a 5% variant allele frequency (VAF). VAF represents the proportion of reads reporting a specific variant relative to the total number of reads covering this gene locus. However, this threshold does not ensure sensitivity for very low VAFs or applications such as minimal residual disease monitoring [9]. Indeed, several studies demonstrated that substantially higher coverage is needed to confidently detect variants with low VAF: for example, Petrackova et al. showed that detection of a 3% VAF with >99% confidence requires approximately 1650× coverage [28], and robust detection of a 2% VAF generally requires a sequencing depth near or above 1000× [27]. Targeted sequencing panels can achieve high sensitivity because selective amplification of specific genomic regions enriches variant-containing loci within the library allowing deeper coverage without excessive sequencing volume [29].

In addition to sequencing depth and VAF, most NGS protocols require a minimum number of supporting reads—that is, individual sequencing containing the variant allele. This requirement is essential to distinguish true variants from sequencing artifacts, particularly at low VAF. In clinical and research practice, thresholds of around 10–30 supporting reads are commonly applied to ensure reliable variant calling and reduce the number of false positives [30,31]. For fusion detection, at least three supporting reads are generally required [30].

### 2.2. NGS Types in Clinical Practice

Whole-genome sequencing (WGS) provides a comprehensive view of genetic variation, capturing both coding and non-coding regions as well as structural variants and copy number alterations, offering unparalleled breadth in genomic analysis. Whole-exome sequencing (WES), in contrast, targets only the protein-coding exons—about 1–2.5% of the genome—providing a more cost-effective approach while still detecting a high proportion of clinically relevant variants [5,32,33].

RNA sequencing (RNA-seq or transcriptome-wide sequencing) is particularly valuable for identifying fusion genes and for capturing gene expression profiles and transcriptomic alterations that may be missed by DNA-based assays [32]. RNA-seq can also be required to define the specific fusion partner of a biologically dominant gene such as in KM2TA rearrangements (e.g., *KMT2A::MLLT3*, *KMT2A::MLLT1*, *KMT2A::ELL*) which carry prognostic significance and cannot be fully resolved with cytogenetic assays, such as FISH, alone [2].

Finally, targeted sequencing panels—the most widely used in clinical diagnostics—focus on a selected set of genes recurrently mutated in a given disease (typically 20–50 genes for MDS and AML), providing higher coverage and analytic sensitivity at reduced cost and with faster turnaround time [10]. By selectively amplifying regions of interest, targeted panels increase the relative representation of relevant sequences within the library reducing the need for very high overall sequencing depth and allowing detection of variants with relatively low VAF [29,33]. Target panels can detect clinically relevant single nucleotide variants (SNV), small insertions and deletions (indels), copy number alterations, fusion genes, and structural variants supporting diagnosis, providing a more precise prognosis definition and guiding therapeutic choices [9]. Notably, a targeted sequencing panel may have a limited sensitivity for detecting large insertions or deletions—such as *FLT3* ITD, *MLL* PTD, and 52 bp *CALR* deletions—which may require more sophisticated bioinformatical approaches or complementary molecular diagnostic assays. This limitation arises from the discrepancy between the large and/or variable size of these alterations and relatively small size of NGS reads (50–300 bp) [16,34,35,36]. However, recent clinical trials suggest that these challenges can be overcome with PCR-NGS-based methods even enabling patient-tailored measurement of *FLT3*-ITD MRD [37]. On the other hand, genomic regions of high GC content, like *CEBPA* variants, may be difficult to amplify during library preparation and may require higher increased sequencing depth to ensure reliable detection [16,38]. However, these problems could also be addressed by using long-read NGS targeted panels designed for AML diagnostics [18]. Recent studies have demonstrated that high diagnostic sensitivity and strong concordance with traditional analytical methods of targeted NGS panels generate target-enriched libraries with improved amplicon specificity making them suitable for accurate *FLT3* ITD and *CEBPA* testing [9,17,36,39].

Most targeted panels have been modified with the addition of new relevant genes according to the most recent WHO/ICC 2022 classifications [40]. The core genes commonly recommended for inclusion in myeloid malignancy panels typically include: *NPM1*, *CEBPA*, *ASXL1*, *BCOR*, *EZH2*, *RUNX1*, *SF3B1*, *SRSF2*, *STAG2*, *U2AF1*, *ZRSR2*, *TP53*, *JAK2*, *CALR*, *MPL*, *CSF3R*, *KIT*, *FLT3*, and *IDH1/2*. Many panels also frequently incorporate *ETV6*, *KRAS*, *NRAS*, and *WT1* [2,17] (Table 1).

However, the numbers of genes (core and/or additional targets) and the specific gene fragments included in targeted panels—such as hotspot mutations, flanking regions, coding or non-coding sequences, or entire gene coverage—can differ substantially between laboratories, particularly in the absence of a standardized NGS approach for AML/MDS diagnosis [26,41,42].

### 2.3. Sample Type and Quantity

**Figure 1:**
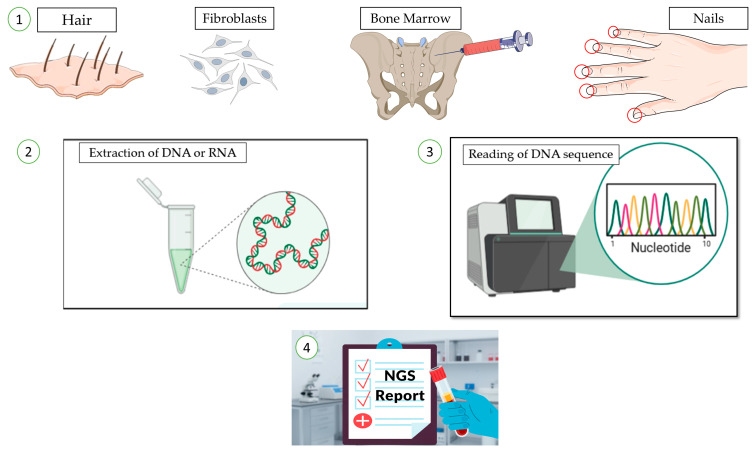
The workflow of NGS, from sample collection to result reporting. (1) Biological specimens, such as hair, fibroblasts, bone marrow, or nails, are first collected from the patient. (2) Nucleic acids (DNA or RNA) are isolated from these samples in the laboratory. (3) The extracted genetic material is then processed and sequenced to determine the precise order of nucleotides. This process generates data that is analyzed by bioinformatics to interpret the genetic sequence. (4) The findings are compiled into a formal laboratory report for clinical assessment.

In the case of most hematological samples, including MDS or AML, tumor cells are obtained from bone marrow or peripheral blood (Figure 1). In myeloproliferative neoplasms, peripheral blood NGS generally shows high concordance with bone marrow results making it a reliable and less invasive diagnostic tool. However, bone marrow samples remain superior to peripheral blood for MRD measurement [43,44]. White blood cell counts or flow cytometry can be used to estimate the number of tumor cells in the sample. When bone marrow aspirate is not available, NGS can also be performed using formalin-fixed paraffin-embedded (FFPE) BM trephine biopsies. However, FFPE samples often yield lower sequencing quality than fresh samples because formaldehyde fixation induces cross-linking between cellular macromolecules and amino groups of DNA bases, reducing DNA sample quality [41]. The required sample amount varies between assays and studies. Illumina recommends around 80 ng of total DNA or RNA per sample, although good results can often be obtained with smaller inputs (40–60 ng) or with higher amounts (100–120 ng) when available [30,31,45]. One study comparing diagnostic panels for *FLT3*-ITD and *CEBPA* testing reported that 200 ng of DNA per sample were required for optimal library preparation [17]. Long-read and ultra-long-read NGS assays may need even larger amounts of DNA, ranging from several hundred nanograms to micrograms [18]. Table 2 summarizes the main NGS tests grouped by specific clinical question.

### 2.4. Results Validation

Due to the complexity of the NGS workflow and the risk of false positives from technical artifacts, bioinformatic errors, or very low-level variants close to the detection threshold, NGS results may require confirmation using other molecular methods like Sanger sequencing, droplet digital PCR, quantitative PCR, or capillary electrophoresis. Current guidelines from the Association for Molecular Pathology (AMP) and the College of American Pathologists (CAP) recommend orthogonal confirmation in selected situations—for example, when results are ambiguous, close to the limit of detection, classified as variants of uncertain significance (VUS), or when they have immediate therapeutic impact [9,46]. For well-validated, high-performance targeted NGS panels, confirmation is often not required for recurrent hotspot mutations, given the high concordance with other molecular diagnostic methods [17,39]. Overall, validation strategies should be adapted to the assay design, the type of variant, and the clinical context, aiming to balance analytical reliability with turnaround time and cost [27].

### 2.5. Standardization

The integration of NGS into the diagnostic workflow for hematologic malignancies underscores the need for standardization across laboratories and platforms. Differences in sequencing chemistry, library preparation, bioinformatics pipelines, and reporting can affect variant detection and interpretation [17,40]. International guidelines from AMP and CAP recommend harmonizing procedures from sample collection and nucleic acid extraction to library preparation, sequencing performance metrics (read length, depth of coverage, uniformity, error rates, variant allele frequency), and post-analytical steps (variant annotation, classification, and reporting) [1,38]. Proficiency testing programs and inter-laboratory comparisons are also used to ensure reproducibility and clinical reliability [9,38]. Although ELN 2022 recommendations for AML, ESMO Clinical Practice Guidelines for MDS, and the WHO 2022 and ICC 2022 diagnostic classifications highlight the integration of NGS into diagnostic and prognostic workflows of AML and MDS and define the minimal set of relevant genes, the insufficient standardization of diagnostic NGS limits its routine implementation in clinical practice [2,3,28,42,47]. Challenges remain in reaching consensus on minimal gene panels, defining limits of detection for clinically relevant variants, and standardizing the use of NGS for measurable residual disease (MRD) monitoring [48]. In addition, existing guidelines published several years ago should be updated to reflect recent technical advances that have since entered into clinical practice. Notably, Italy has recently issued specific SIES guidelines addressing the role of advanced molecular diagnostics, including NGS, in adult AML, and similar recommendations have been released in other countries [49,50].

### 2.6. Type of Alterations

NGS enables the detection of single nucleotide variants (SNVs), small insertions and deletions (indels), and, depending on the platform, copy number variations (CNVs) and structural rearrangements. RNA-based sequencing can also detect fusion transcripts, which are particularly relevant in AML.

It is important to use standardized and consistent nomenclature to ensure clear communication and effective data sharing. The Human Genome Variation Society (HGVS) provides widely accepted guidelines for variant naming, and their use is recommended in clinical reporting. Reports should specify the reference sequence used, along with the coding and protein nomenclature, to facilitate functional interpretation. For coding variants, the longest validated reference transcript for each gene should be used and clearly indicated.

Commonly used reference databases include RefSeq (“https://www.ncbi.nlm.nih.gov/refseq (accessed on 20 October 2025)”), Ensembl (“http://www.ensembl.org/index.html (accessed on 20 October 2025)”), and Locus Reference Genomic (LRG; “https://www.lrg-sequence.org (accessed on 20 October 2025)”) [46].

### 2.7. Result Interpretation

Bioinformatic analysis can be divided into three main stages:

- Primary Analysis:

Fluorescent signals generated during sequencing are converted into text files (FASTQ) representing the sequence of individual bases.

- Secondary Analysis:

Sequences from multiple samples run together are separated according to their indices in a process called demultiplexing. The reads are then aligned to a reference genome, and differences are detected in a step known as variant calling.

This process can identify thousands of variants, which are collected into a single VCF (“Variant Call Format”) file for each patient.

- Tertiary Analysis:

The final stage involves interpreting the identified variants in the clinical context, evaluating their potential relevance and pathogenicity, and determining possible causal relationship with the patient’s disease.

Variants can be classified according to their actionability or pathogenicity.

Currently, two main systems are used to classify the clinical significance of somatic variants: the AMP/ASCO/CAP tier system and the ClinGen/CGC/VICC system.

In the first one, somatic variants are grouped into four tiers according to their clinical relevance for cancer diagnosis, prognosis, and treatment: Tier I includes variants with strong clinical significance; Tier II comprises variants with potential clinical significance; Tier III refers to variants of uncertain clinical significance; and Tier IV includes variants that are benign or likely benign [46].

The second classification is based on a combined assessment of multiple criteria, including population frequency data, computational or predictive algorithm results, and whether the variant affects the important functional domains of the protein. Importantly, no single criterion is sufficient to determine pathogenicity of a variant on its own; instead, pathogenicity must be supported by multiple lines of evidence. This integrated approach enables the classification of variants into five categories: 1—benign; 2—likely benign; 3—variant of uncertain significance; 4—likely pathogenic; and 5—pathogenic.

**Figure 2:**
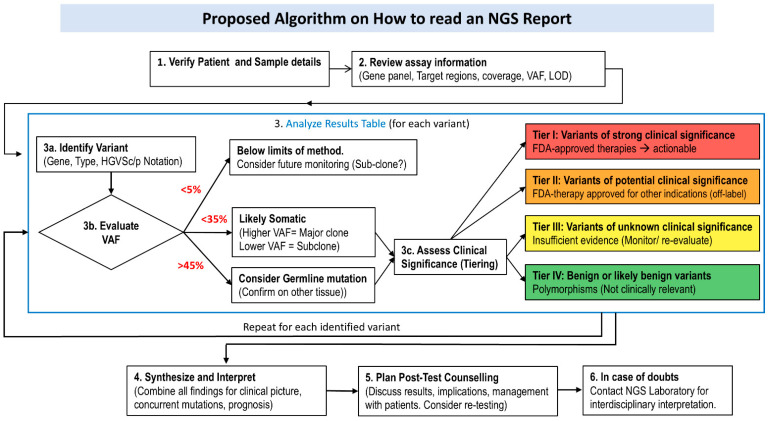
Proposed algorithm for the interpretation of next-generation sequencing (NGS) reports in hematology. The flowchart outlines a stepwise approach, starting from the verification of pre-analytical variables and technical assay parameters (limit of detection, coverage). The core analysis focuses on variant evaluation based on variant allele frequency (VAF) and potential germline origin. Variants are stratified into four tiers of clinical significance (Tier I–IV, AMP/ASCO/CAP Guidelines) to guide therapeutic decision-making and post-test counseling.

Based on the strength and extent of available scientific evidence, pathogenicity criteria are further ranked as oncogenic very strong (OVS1), strong (OS1-3), moderate (OM1-4), or supporting (OP1-4). Conversely, benignity criteria are classified as somatic benign very strong (SBVS1), strong (SBS1-2), or supporting (BP1-2) [51]. In Figure 2 we visualized a proposed practical algorithm on “How to read an NGS report”.

### 2.8. Useful Database

With the increasing number of large-scale genome sequencing projects across various cancer types, including hematological malignancies, a substantial amount of data has been generated and incorporated into multiple databases.

The clinical and biological relevance of a given variant is closely associated with its recurrence in cancer contexts. Therefore, it is essential to evaluate the incidence and prevalence of tumor variants identified in different tumor entities, using databases and published literature. For the annotation of somatic variants, several specialized cancer databases are available, including the Cancer Gene Census (“http://cancer.sanger.ac.uk/cancergenome/projects/census/ (accessed on 21 October 2025)”), the Catalogue of Somatic Mutations in Cancer (COSMIC; “http://cancer.sanger.ac.uk/cancergenome/projects/cosmic/ (accessed on 21 October 2025)”), and The Cancer Genome Atlas (TCGA; “http://cancergenome.nih.gov/ (accessed on 21 October 2025”)). These databases provide information on sequence variants in different cancer types and subtypes, cross-references to other genomic databases, as well as including references to published literature, cellular pathways, targeted therapies, clinical trials, and outcome data [51].

Probable germline variants that may reside in genes associated with cancer predisposition syndromes can be detected during the sequencing of the tumor sample. Nevertheless, it is important to correlate this data with tumor cellularity [46].

In this regard, it may be useful to consult specialized databases, such as Online Mendelian Inheritance in Man (OMIM, “http://www.ncbi.nlm.nih.gov/omim (accessed on 21 October 2025)”) and Human Gene Mutation Database (HGMD, “http://www.hgmd.cf.ac.uk/ac/index.php (accessed on 21 October 2025)”), that provide information helpful for evaluating the potential nature of these variants. Among these, ClinVar (“http://www.ncbi.nlm.nih.gov/clinvar (accessed on 21 October 2025)”) is widely used, cataloging germline variants across various tissues and classifying them from pathogenic to benign. ClinVar also provides supporting clinical evidence, associated studies, and expert panel classifications, and occasionally reports somatic occurrences for certain variants [52].

When the germline nature of a variant is suspected, confirmation via a validated germline test is appropriate. This should be performed on a sample of tissue not affected by the disease for which the somatic analysis was initially requested following consulting with the physician and after obtaining a properly completed and signed informed consent form from the patient.

Therefore, it is recommended that reports explicitly state that the test cannot reliably distinguish somatic variants from germline or benign ones, in cases where a germline origin is suspected [46].

To evaluate the frequency of variants in large populations, population databases are used. These resources provide information on specific alleles at defined loci in large cohorts, often stratified by distinct subpopulations. When using these databases, it is necessary to consider whether the cohorts included healthy or affected individuals, whether multiple individuals from the same family were included, and the age range of the subjects. Population databases are frequently used to characterize benign variants, based on an arbitrary cut-off (minor allele frequency, MAF). There is no standardized MAF threshold: a threshold of 1% is commonly recommended and is potentially adjusted according to the patient’s specific ethnicity. Among the most widely used population databases are the following: 1000 Genomes Project (“http://browser.1000genomes.org (accessed on 21 October 2025)”), Exome Variant Server (“http://evs.gs.washington.edu/EVS (accessed on 21 October 2025)”), dbSNP (“http://www.ncbi.nlm.nih.gov/snp (accessed on 21 October 2025)”), dbVar (“http://www.ncbi.nlm.nih.gov/dbvar (accessed on 21 October 2025)”), and ExAC (“http://exac.broadinstitute.org/ (accessed on 21 October 2025)”) [46].

Finally, when evaluating a variant in a gene, in silico prediction algorithms are commonly employed to assess its potential impact on splicing and on the structure and function of the encoded protein. These algorithms typically consider several key factors including the evolutionary conservation of the amino acid or nucleotide residue, the biochemical consequences of amino acid substitution based on physicochemical properties, and the position of the variant within the translated protein.

Some of the most used in silico tools are PolyPhen2 Ver. 2.2.3 (http://genetics.bwh.harvard.edu/pph2 (accessed on 21 October 2025)), SIFT or PROVEAN v.1.1.3 (http://sift.jcvi.org (accessed on 21 October 2025)), MutationTaster v. GRCh37/Ensembl 69 (http://www.mutationtaster.org (accessed on 21 October 2025)), and CADD v1.6 (http://cadd.gs.washington.edu (accessed on 21 October 2025)).

It is important to emphasize that these tools are predictive, and should be used with caution when interpreting sequencing variants as the sole evidence for clinical decision-making [46].

### 2.9. Report Structure

At the end of the NGS analysis process, it is essential to prepare a comprehensive report summarizing the results. This report should be thorough, providing the necessary technical details of the methods used, while remaining understandable to non-specialist healthcare professionals and patients, and concise enough to avoid overwhelming the reader with excessive information. The report must include information about the laboratory that performed the analysis, the patient’s personal details, the sample’s traceability, the clinical question addressed, the variants identified along with their interpretation, and the methods employed for the analysis.

It is important that the report clearly displays the name of the laboratory with its certification (e.g., UNI EN ISO 9001) [53] and accreditation (e.g., UNI EN ISO 15189 [54], UNI CEI EN ISO/IEC 17025 [55]). Preferably, the report should include the name of the director, the laboratory’s contact information, as well as the name of the referring clinician and the department of origin, ensuring clarity and traceability of responsibility [22].

It is important that the report includes the patient’s personal information, such as surname, first name, date of birth, gender, and ethnicity.

The report must also provide details about the biological sample, including a unique identifier, date of collection, and sample type (e.g., peripheral blood, bone marrow) and, whenever possible, additional information such as an estimate of the percentage of blasts, the diagnosis, and/or the clinical indication for the test.

The report must include detailed information on the NGS assay used, specifying sequencing technology or platform, the panel name and version, as well as a list of the analyzed genes and the covered regions.

A concise description of the methodology and its limitations should also be provided and should include a list of all software tools used to generate the results with their version numbers, as well as the reference genome employed (GRCh37-hg19 or GRCh38) [56].

It is important to indicate the limitations of the method: specifying which types of variants the approach cannot detect, which alterations will not be reported, their potential oncogenicity/benignity, or clinical actionability. The report may include the overall coverage achieved for each sample, measured as the average coverage over the entire sequenced genomic region. According to AMP/CAP guidelines, an average coverage of at least 250× is recommended to reliably detect somatic variants [9]. In this context, a VAF threshold of 5% is commonly used for variant calling and should be explicitly stated in the report [22].

The results section is the central component of the report. For clarity, it may be preferable to present the variants in order of clinical relevance, with the most pathogenic or actionable variants listed first.

**Figure 3:**
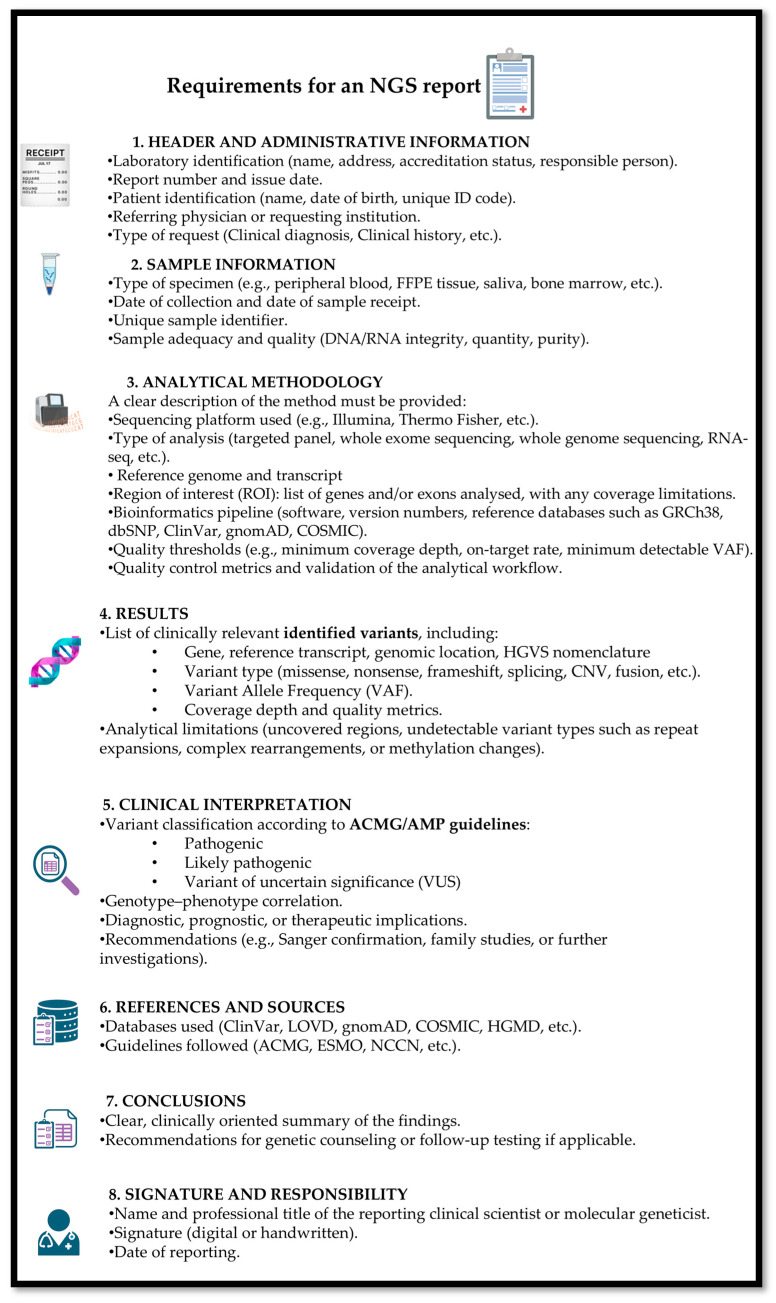
This vignette shows the advised requirements for an effective NGS report.

All reported variants must be described using HGVS.c and HGVS.p notation, together with their VAF and classification. The identified variants should be summarized in a dedicated section, structured to ensure immediate understanding, and supplemented with references to relevant literature, when appropriate [46]. The report concludes with the date of reporting, the full name of the biologist who performed the analyses, and the signature of the laboratory manager. Each page of the report must be appropriately numbered [6] (Figure 3).

### 2.10. NGS vs. Quantitative-PCR vs. Digital-PCR

Today, laboratories have access to a variety of technologies that can be used individually or in combination to achieve their experimental objectives. These include long-established methods, such as quantitative PCR (q-PCR), as well as more recent techniques, such as digital PCR (dPCR) and NGS.

The qPCR allows real-time measurement of DNA amplification through fluorescence monitoring, providing either relative or absolute quantification.

In dPCR, the sample is divided into thousands of independent partitions: the ratio of positive partitions, those showing a fluorescent signal, to the total number of partitions is used to calculate the concentration of the target in the sample. This approach allows absolute quantification, enabling the detection of a very low number of target molecules without interference from PCR inhibitors and without the need for a standard curve, making it a sensitive and accurate technique [57,58]. Although qPCR has a more limited resolution, it remains a faster, and a more cost-effective method, suitable for the simultaneous analysis of multiple samples and targets. Both qPCR and dPCR require prior knowledge of the mutation of interest, which allows for longitudinal monitoring over time.

NGS, in contrast, supports large-scale quantitative and qualitative analyses, enabling simultaneous investigation of multiple mutations across numerous samples. This approach generates analysis with a larger and more complex datasets, with increased costs and reporting times, and typically it has lower sensitivity than dPCR [59,60].

## 3. NGS and Impact on Diagnosis and Classification

### 3.1. NGS Role in MDS: From Diagnosis to Classification

The transition from the previous WHO2016 to the updated ICC 2022 and WHO 5th editions was marked by the introduction of new genetically defined MDS categories [2,47,61,62,63]. In this context, the availability of NGS testing is crucial not only to correctly identify patient category but also to address patients to genetically driven therapies. Among the major innovations of the WHO 5th edition is the definition of MDS with low blast count and SF3B1 mutations, which has been incorporated into the former category of MDS LB with ring sideroblasts (RS) [2]. This new category has also been adopted by the ICC classification, replacing the previous MDS with RS [47]. WHO-2022 has also introduced another genetically driven category defined as MDS with bi-allelic *TP53* mutations, independent of blast count [47]. Similarly, ICC introduced the diagnostic categories of MDS and MDS/AML with mutated *TP53*, which are distinguished on the basis of blast percentage [2]. The new “MDS/AML” category represents an overlap group that includes cases with 10 to 19% blasts, replacing the former WHO category of MDS with excess-blast 2. This change better reflects the biological continuum between MDS and AML [62]. NGS is essential for defining MDS/AML, as several mutations must be either identified or excluded to ensure accurate classification. In particular, MDS/AML cases should not harbor AML-defining genetic abnormalities such as *NPM1* or *CEBPA*. The ICC further subdivides MDS/AML into (i) MDS/AML with mutated *TP53*, (ii) MDS/AML with myelodysplasia-related gene mutations or cytogenetic abnormalities, and (iii) MDS/AML, NOS (not otherwise specified). Importantly, correct classification requires mutational assessment of several genes, including *TP53*, *ASXL1*, *BCOR*, *EZH2*, *SF3B1*, *SRSF2*, *STAG2*, *U2AF1*, *ZRSR2*, and *RUNX1* [47]. Finally, both the WHO 2022 and the ICC classifications introduced relevant updates regarding clonal hematopoiesis, including the formal recognition of Clonal Hematopoiesis of Indeterminate Potential (CHIP) and clonal cytopenia of undetermined significance (CCUS) as part of the spectrum of myeloid neoplasms [2,47].

### 3.2. CHIP and CCUS, the Role of NGS

CHIP is currently defined by the presence of somatic mutations commonly seen in hematologic malignancies (e.g., *DNMT3A*, *TET2*, *ASXL1*) in individuals without cytopenia or dysplasia. This condition is increasingly detected through NGS in otherwise healthy individuals and is more common in older adults [64,65]. Several studies have demonstrated that individuals with CHIP have an increased risk of developing hematologic malignancies, especially MDS and AML, as well as a higher risk of atherosclerotic cardiovascular disease [66,67,68]. Variant allele frequency ≥ 2% is generally considered significant for CHIP. While CCUS is not recognized as a diagnostic category in WHO 2022, the ICC classification defines it as a distinct diagnostic entity, requiring close clinical follow-up, particularly when high-risk mutations like *TP53* are detected [2,47,69]. According to the ICC, CCUS is defined as persistent unexplained cytopenia in at least one lineage, in the absence of morphologic dysplasia or increased blasts [47]. NGS is essential for this diagnosis, as it enables detection of at least one somatic mutation in genes commonly mutated in myeloid neoplasms (e.g., *DNMT3A*, *TET2*, *ASXL1*, *SF3B1*, *SRSF2*, etc.). The type of mutations and VAF are particularly relevant in these conditions. CHIP is typically asymptomatic and carries a 1% annual risk of progression to hematologic malignancy. However, multiple studies have identified several high-risk features in CHIP, including specific mutations (*TP53*, *U2AF1*, *SRSF2*, *IDH1/2*), the presence of multiple concomitant mutations, detection of larger clones (e.g., VAF 10–15%) or progressive clonal expansion over time [69,70]. In CCUS, mutations in spliceosome genes and epigenetic regulators are associated with a higher risk of evolution when compared to typical CHIP mutations. Additionally, the presence of ≥2 pathogenic mutations confers a ~50% risk of progression within 5 years [71].

### 3.3. Novel Genetically Based Categories in MDS

#### 3.3.1. SF3B1 in MDS

*SF3B1* (splicing factor 3b subunit 1) is the largest subunit of the SF3B complex and serves as a core component of the U2 snRNP, where it plays a key role in branch site recognition in the early stages of spliceosome assembly. Mutations in SF3B1 were originally identified in 2011 and are observed across all the spectrum of myeloid malignancies, including MDS, MDS/AML, and AML [72]. However, they are particularly enriched in MDS and especially in MDS with ring sideroblasts, where up to 65% of patients carry a *SF3B1* mutation [72,73]. Several studies have confirmed that *SF3B1* mutations are detected in over 90% of MDS cases with ≥5% ring sideroblasts. Most *SF3B1* mutations are heterozygous missense variants clustered in exons 12–15, with K700E representing approximately one-third of the variants. These mutations alter protein function but generally preserve its structural integrity [73]. According to the ICC classification, MDS with *SF3B1* mutation is now recognized as a distinct diagnostic entity and no longer requires the presence of ring sideroblasts [47]. To meet diagnostic criteria, MDS patients with *SF3B1* mutations must have <5% in the bone marrow and <2% in the peripheral blood and must not carry specific cytogenetic and molecular abnormalities, including isolated del(5q), −7/del(7q), abn3q26.2, or complex karyotype, *TP53* multi-hit, or *RUNX1* mutations [47]. The WHO 2022 is more conservative and still maintains the entity MDS with low blasts and ring sideroblasts in cases with wild-type *SF3B1* and ≥15% ring sideroblasts [2]. This category represents a genetically homogeneous group and is associated with low progression rates and better overall survival (OS) when detected in patients with low blast counts and without additional genetic alterations [73].

#### 3.3.2. TP53 in MDS

TP53 is a tumor suppressor gene located on chromosome 17p13.1 and is among the most frequently mutated genes in human cancers where its inactivation is typically associated with poor clinical outcomes. In MDS, pathogenic *TP53* alterations are identified in 7–11% of cases. Importantly, *TP53* inactivation in MDS is not limited to point mutations but may also involve additional mechanisms of loss of genetic material, including copy-neutral loss of heterozygosity and segmental deletions, which further contribute to loss of tumor suppressor function [74,75]. Most *TP53* mutations (−80%) are missense substitutions involving exons 5 to 8, resulting in amino acid changes, while the remaining cases include truncating alterations. Physiologically, active p53 regulates the transcription of more than 150 genes implicated in DNA damage repair, apoptosis, senescence, and cell cycle arrest. Loss-of-function mutations lead to non-functional p53 protein, impair genomic stability, disrupt hematopoietic stem cell self, and promote uncontrolled proliferation. In the WHO 2022 classification, a distinct diagnostic category termed “MDS with biallelic *TP53* inactivation (MDS-biTP53)” has been introduced, defined by the presence of multiple *TP53* alterations leading to complete loss of p53 function [2]. Biallelic *TP53* (biTP53) alterations may consist of multiple mutations, mutations with a VAF > 50%, or mutations with concurrent deletion of the other allele (*TP53* copy number loss or copy-neutral loss of heterozygosity). The detection of biallelic inactivation of *TP53* is complex and requires more than one technique. NGS analysis is essential to detect single mutation or multiple mutations in the gene, covering at least exons 4 to 11 [76]. The first-line method for detecting loss of genetic material at the *TP53* site is fluorescence in situ hybridization with specific probe sets for the *TP53* locus on 17p13.1. NGS, instead, can contribute to the identification of multi-hit *TP53* alterations. In particular, the presence of more than one *TP53* mutation typically suggests involvement of both alleles, indicating biallelic inactivation; secondly, detection of *TP53* mutations with VAF ≥ 50% can be used as a surrogate (although not certain) for loss of the second *TP53* allele [2]. Thus, NGS complements FISH by providing mutational data that supports the recognition of biallelic *TP53* inactivation. At the same time, NGS panels may fail to detect 17p deletions present in low cell fractions, which are more easily identified by FISH, explaining the discrepancy between *TP53* alteration detection by both methods and supporting their complementary use [77,78]. The ICC classification has introduced two distinct categories of MDs with *TP53* mutations: MDS with mutated *TP53* and MDS/AML with mutated *TP53*. The first includes MDS cases with blasts up to 9% with either multi-hit *TP53* mutation or *TP53* mutation (VAF > 10%) and complex karyotype often accompanied by loss of 17p [47]. Multi-hit *TP53* mutations include the presence of either two distinct *TP53* mutations (each VAF > 10%), a single TP53 mutation with 17p deletion or copy-neutral LOH at the *TP53* locus, or a *TP53* mutant VAF of >50% [47]. MDS/AML with mutated *TP53* is defined as cases with 10–19% blasts and any *TP53* mutation with VAF > 10% [47]. Overall, these *TP53*-mutated MDS categories, particularly multi-hit *TP53* are often associated with complex karyotype and poor prognosis [79].

### 3.4. Impact of NGS in the Definition of AML, Novel Genetic Classifications

Nowadays, AML can be classified according to two systems: the WHO fifth edition [2] or the International Consensus Classification (ICC) of myeloid neoplasms and acute leukemias [47]. Genetic features have now surpassed morphologic and cytometric features, firmly establishing NGS analysis as a cornerstone in AML diagnosis. The ICC classification has strengthened the role of genetics by lowering the blasts threshold to 10% for cases harboring recurrent genetic abnormalities (*BCR::ABL1* excluded). In addition, the ICC has introduced the MDS/AML category in cases with 10 to 19% of blasts. The ICC approach can be viewed as hierarchical. In the first step, recurrent genetic abnormalities, NPM1 mutations, and in-frame bZIP domain mutations of *CEBPA* should be investigated, and if none are present the second step requires assessment for *TP53* mutations (VAF  >  10%) and myelodysplasia-related gene mutations or cytogenetic abnormalities. Therefore, the second step of the ICC relies almost entirely on NGS for both identification and quantification of *TP53* mutations and for detecting mutations in *ASXL1*, *BCOR*, *EZH2*, *SF3B1*, *SRSF2*, *STAG2*, *U2AF1*, *ZRSR2*, and *RUNX1*, which are myelodysplasia-related genes [57].

#### 3.4.1. TP53 Mutations in AML [58]

In de novo AML, *TP53* mutations are detected in 5–10% of cases and are more frequent in elderly patients and in therapy-related AML. Similarly to MDS, *TP53* mutations may be present in either monoallelic or biallelic form; in the latter condition, the inactivation of the second allele may occur through additional point mutations, copy-neutral LOH (cnLOH), or loss of heterozygosity (LOH). *TP53* mutations define an aggressive subset of AML, regardless of whether the disease is de novo, evolves from a preceding MDS, or is therapy-related, and they are frequently associated with a complex karyotype. Unlike in MDS, in the ICC classification, a single *TP53* mutation with VAF > 10% is sufficient to define AML with *TP53* mutations (>20% blasts) or MDS/AML with *TP53* mutation (10–19%) [2,47].

#### 3.4.2. MDS-Related Mutations

The ICC classification, as well as theELN2022 guidelines, has introduced a nine-gene signature (*ASXL1*, *BCOR*, *EZH2*, *RUNX1*, *SF3B1*, *SRSF2*, *STAG2*, *U2AF1*, and *ZRSR2*) to define the new category of AML (>20% blasts) or MDS/AML (10–19% blasts) with myelodysplasia-related mutations [2,47]. These mutations were classified as adverse risk in the ELN 2022 due to their strong association with secondary AML arising from prior MDS or chronic myelomonocytic leukemia (CMML) [80]. In the ICC classification, this category is incorporated alongside MDS-related cytogenetic abnormalities, thus expanding the former WHO 2016 category of AML with myelodysplasia-related changes, which was based solely on morphologic assessment [47,62]. The WHO 5th edition also recognizes the category of AML, myelodysplasia-related WHO-AML-MR defined by specific molecular and cytogenetic abnormalities, while still including cases of AML arising from MDS or MDS/MPN [2]. Notably, the WHO 5th edition includes only eight of the nine genes used in the ICC classification, excluding *RUNX1*. These eight genes have been demonstrated to maintain over 95% specificity for identifying secondary AML evolving from prior MDS, thus helping to distinguish s-AML from de novo AML [1]. More specifically, *SRSF2*, *SF3B1*, *U2AF1*, and *ZRSR2* are mutated in nearly half of s-AML cases and encode spliceosome components critical for RNA processing, particularly for pre-mRNA splicing. Most *SRSF2* mutations affect the P95 residue, whereas *SF3B1* mutations predominantly occur at K700, with a smaller proportion involving K666. *U2AF1* gene also shows well-defined mutational hotspots at S34F and Q157. In contrast, *ZRZS2* mutations are scattered throughout the gene but occur most frequently within the Pre-ZF1 (49%), UHM (27%), and Post-ZF2 (13%) domains [81,82]. *ASXL1*, *EZH2*, and *BCOR* belong to the class of chromatin modifiers. ASXL1 regulates epigenetic programs and transcription through interaction with Polycomb complex proteins and various transcription activators and repressors. This gene is ubiquitously mutated in myeloid neoplasms, with most variants occurring in the final exon (exon 13). One of the most common alterations is the frameshift mutation G646Xfs*12 [81,83]. EZH2 is a lysine methyltransferase and represents the central core protein of the Polycomb Repressive Complex 2 (PRC2). Most mutations cluster in the SET ([Su(var)3-9, Enhancer-of-zeste and Trithorax]) or CXC (cysteine-rich region) domain at the C-terminus of the protein, regions that are essential for its methyltransferase activity. Approximately half of the variants are stop-gain or frameshift mutations, causing premature truncation of the protein [84]. BCOR is a transcription factor involved in embryogenesis, mesenchymal stem cell function, hematopoiesis, and lymphoid development. It acts as a tumor suppressor, and pathogenic variants may occur throughout the gene, including nonsense, frameshift, missense, or splice-site mutations [85]. STAG2 is a core component of the cohesin complex essential for chromatin organization, transcriptional regulation, and DNA repair. Mutations are scattered throughout the gene, although most are located in the N-terminus and in the STAG domain, and are typically nonsense, frameshift, or splice-site [86]. The identification of MDS-related mutations by NGS has relevant clinical implications since it can identify a subgroup of high-risk AML patients who may benefit from alternative frontline treatment (e.g., CPX351 [87]) and subsequent consolidation with allogeneic hematopoietic stem cell transplantation (allo-HSCT).

#### 3.4.3. *RUNX1* Mutations

*RUNX1* mutations are enriched in s-AML and the ICC classification included this gene among the defining features of AML with MDS-related mutations. *RUNX1* encodes a transcription factor that is a key regulator of hematopoiesis involved in hematopoietic stem cell emergence and function. The mutational spectrum consists mainly of missense mutations in the N-terminal region, particularly within the RUNT domain, and truncating mutations in the C-terminal portion of the protein within the transactivation domain (TAD). *RUNX1* mutations are consistently associated with poor outcomes in all myeloid malignancies, including AML [88].

#### 3.4.4. AML-Defining Mutations

*CEBPA*. The *CEBPA* gene, located on chromosome 19q13.11, encodes a transcription factor essential for myeloid differentiation. From 5 to 15% of AML cases harbor *CEBPA* mutations that are more frequent in younger adults [89]. These mutations can affect both the N-terminal TAD and the C-terminal bZIP domain, and can be either monoallelic or biallelic [89]. Both the ICC and WHO 2022 classifications recognize *CEBPA*-mutated AML as a distinct entity, but differ in scope: the ICC includes only AML cases with in-frame bZIP domain mutations (allowing a lower blast count ≥10%) [2,47]. Conversely, WHO defines a broader category of AML with *CEBPA* mutations (including both biallelic and bZIP monoallelic mutations). *CEBPA* mutations can be detected by PCR, but NGS represents a useful tool which enables to identify both the number of mutations (mono vs. biallelic) and their location across the N-terminal and the C-terminal regions [89]. This distinction is clinically relevant since most familiar CEBPA mutations are located in the N-terminal portion and are often accompanied by a “second-hit” in the C-terminal domain [90]. Detection of such variants with a VAF ≥ 30–40% should prompt germline testing. Some studies have also highlighted challenges for NGS in detecting biallelic *CEBPA* mutations, partly due to the high GC content of the gene [59].

*NPM1*. The nucleophosmin (NPM1) gene encodes the most abundant nucleolar protein. Through its role as a histone chaperone and its ability to shuttle between the nucleus and cytoplasm, NPM1 participates in multiple cellular processes, including nucleolus formation via liquid–liquid phase separation, regulation of ribosome biogenesis and transport, control of DNA repair and centrosome duplication, and response to nucleolar stress. *NPM1* is the most frequently mutated gene in AML occurring in about 30–35% of cases [91,92]. Due to its unique biological and clinical features, *NPM1*-mutated AML is recognized as a distinct entity in both the WHO 5th edition and ICC classifications of myeloid malignancies [2,47]. Mutations in *NPM1* atypically affect the C-terminal region, leading to its abnormal localization in the cytoplasm of the leukemic cells [91]. A new class of drugs, the Menin inhibitors (e.g., revumenib), has shown activity in relapsed/refractory AML [93,94,95]. Several studies have compared detection of *NPM1* by PCR and NGS [96,97,98], highlighting some differences. In practice, PCR offers faster turnaround times than NGS and high sensitivity suitable for MRD monitoring. NGS, on the other hand, allows detection of rare or atypical mutations beyond the common hotspot regions [99,100].

### 3.5. AML Mutations Driving Target Therapies (IDH1/2 and FLT3)

At diagnosis of AML, mutations in *FLT3* and *IDH1* or *IDH2* represent actionable targets for patients eligible and ineligible for intensive chemotherapy (IC), respectively [101]. Detecting these mutations is therefore essential; PCR remains the gold standard (e.g., *Abbott RealTime IDH1* [102]) due to its rapid turnaround time and lower costs. Clinically, we can add midostaurin to the standard 7 + 3 backbone in the presence of *FLT3*-ITD or *FLT3*-TKD, while quizartinib is used specifically for *FLT3*-ITD [103,104]. Similarly, in patients ineligible for IC, *IDH1* positive cases can benefit from the addition of ivosidenib to the azacitidine backbone [105]. NGS shows good concordance with PCR, although its sensitivity is slightly lower for *FLT3*-ITD [98].

## 4. NGS and Impact on Risk Stratification

When hematologists evaluate a new patient with AML or MDS, one of the first questions is “What is the prognosis of this neoplasia?”. Several prognostic tools have been developed to answer this key question, based on age, comorbidity, geriatric score, fitness of the immune system, genetic features of the disease, and effectiveness of available therapies play a critical role [106,107,108]. Earlier prognostic systems also relied on cytogenetical data (e.g., ELN2017) [109], but more recent risk stratification incorporates multiple genetic alterations, that require NGS for detection. Therefore, NGS analysis is increasingly considered essential at diagnosis of AML or MDS. Initial stratification also guides therapeutic decisions such as indication for allo-HSCT. In this section, we will focus on the impact of NGS results on risk stratification.

### 4.1. Myelodysplastic Syndromes: Prognostic Impact of Molecular Data

MDS represent both a diagnostic and classification challenge, mainly due to their genetic complexity. Somatic mutations are increasingly incorporated into modern WHO and ICC classifications, paralleling efforts to improve traditional risk stratification tools by including the mutational profile of the most frequently altered genes in MDS [110]. MDS is a heterogeneous disease and accurate survival prediction is essential for therapeutic decision-making. Traditionally, the IPSS-R has been the most widely used risk tool to predict outcomes, incorporating cytopenias, bone marrow blast percentage, and cytogenetic abnormalities [111]. IPSS-R has been widely validated [112], however disease evolution can vary considerably, depending on both disease- and patient-related factors. In recent years, our understanding of the genetic landscape of MDS has expanded and multiple studies have examined the impact of somatic mutations on patient outcomes. Based on data from 3711 patients with MDS profiled for mutations in 152 genes, the IPSS-M is a recently developed risk stratification score that integrates molecular information with traditional parameters, including cytopenias, bone marrow blast percentage, and cytogenetics. MDS patients are classified into six categories based on the mutational status of 31 genes commonly included in routine NGS myeloid panels, divided into 16 main effect genes and 15 residual genes. Multi-hit *TP53*, *FLT3*-ITD/TKD, and *KMT2A*-PTD are particularly influential in predicting adverse outcomes [113]. Interestingly, 46% of MDS patients were reclassified by IPSS-M compared with their original IPSS-R categories, with the majority being assigned to higher risk groups. IPSS-M has been validated in large patient cohorts for its prognostic accuracy; however, data on how this reclassification should guide therapeutic decisions is still limited [114,115,116].

In these cohorts, 20% of patients classified as low-risk by IPSS-R were upstaged to intermediate-risk by IPSS-M, and 25% of intermediate-risk IPSS-R patients were reclassified into higher-risk IPSS-M groups. Notably, a similar proportion of intermediate-risk IPSS-R patients were down staged to low-risk categories under IPSS-M. In clinical practice, however, re-stratification does not necessarily translate into major treatment changes for all affected MDS patients [114,115,116]. A recent analysis by Zamanillo et al. showed that IPSS-M reclassification would potentially influence treatment decisions in 22.2% of patients: 17% would become candidates for treatment intensification and 5% for de-escalation. Yet, only 6.6% of the overall cohort would actually qualify for higher-intensity therapy when age and fitness were considered [117]. In the context of HSCT, Tentori and colleagues reported that an IPSS-M-based strategy favored delayed transplantation for patients < 70 years with low or moderate-low risk, whereas immediate transplantation improved life expectancy in those with moderately high, high, or very high risk [118]. Their findings indicated that 15% of patients originally considered for immediate HSCT by IPSS-R would benefit from delayed HSCT under IPSS-M, and 19% of those initially assigned to a delayed strategy by IPSS-R would instead benefit from immediate HSCT when reassessed by IPSS-M. Overall, an IPSS-M-based policy yielded a significant gain in life expectancy compared with IPSS-R (*p* = 0.001). To date, the lack of prospective clinical trials using IPSS-M limits its broad implementation for systematic MDS risk stratification. Nonetheless, the molecular information integrated into IPSS-M is likely to assist treatment decision-making, particularly for transplant-eligible younger patients (<70 years). Considering the entire MDS population, the proportion of patients who would undergo a meaningful therapeutic change based on IPSS-M remains relatively small, under 10% in older adults and under 19% in younger individuals. For these patients, molecular findings must be incorporated into a comprehensive clinical assessment that includes fitness, transplant eligibility, HLA matching, and donor-related factors.

### 4.2. Acute Myeloid Leukemia

ELN recommendations for AML risk stratification have evolved since the 2010 edition, progressively including the growing evidence on the prognostic impact of gene mutations. Compared with the 2010 and 2017 editions, the most recent 2022 update requires mutational analysis of 13 genes to accurately assign AML patients to favorable-, intermediate- or adverse-risk categories. In particular, the adverse-risk group has been expanded to include seven additional mutations: *BCOR*, *EZH2*, *SF3B1*, *SRSF2*, *STAG2*, *U2AF1*, and *ZRSR2*—together with *ASXL1* and *RUNX1* (already included in ELN 2017)—forming the “myelodysplasia-related” (MR) gene group [6,7]. Therefore, the use of NGS has become essential for correct and accurate risk stratification in AML.

The presence of any of these mutations allocates patients in the adverse-risk category unless accompanied by favorable-risk alterations. TP53 mutations are particularly emphasized in ELN2022. Several studies have validated the ELN2022 guidelines and further explored the role of MR mutations. Most studies highlight significant heterogeneity in outcomes among patients with different MR mutations: for example *RUNX1*, *ASXL1*, and *U2AF1* are generally associated with worse outcomes, whereas *EZH2*, *STAG2*, and *ZRSR2* tend to show intermediate-risk outcomes [119]. The evolving AML treatment landscape has led to the development of low-intensity regimens for older or unfit AML patients [120]. Hypomethylating agents (HMA), azacitidine and decitabine, as well as HMA-based combination therapies with venetoclax or ivosidenib in *IDH1*-mutated AML, have improved outcomes in these patient categories [105]. However, previous risk stratifications, including ELN 2022, were based on clinical trials of intensively treated patients and the attempt to apply this classification to lower-intensity regimens has been shown to be unsatisfactory.

#### Stratification of AML for Patients Treated with Less-Intensive Therapy

In 2024, ELN proposed a genetic-based risk classification (2024 ELN Less-Intensive) for patients with newly diagnosed AML receiving lower-intensity regimens based on HMAs [121]. This classification was derived from large trial cohorts, including ASTRAL-1 randomizing guadecitabine or other therapies (guadecitabine vs. azacitidine, decitabine, or low-dose cytarabine (LDAC)) [122], VIALE-A trial (NCT02993523) randomizing the combination of azacitidine and venetoclax versus azacitidine and placebo, and phase 3 AGILE study (NCT03173248) adding IDH1-inhibitor ivosidenib to azacytidine (compared to azacitidine-placebo). Karyotype status together with a small set of genes, including *NPM1*, *IDH1/2*, *FLT3*-ITD, *DDX41*, *NRAS*, *KRAS*, and *TP53*, was sufficient to stratify patients into favorable-, intermediate-, and adverse-risk categories. The intermediate-risk group in ELN 2024 remains still quite broad, as many cytogenetic and molecular abnormalities lack solid prognostic data in the context of lower-intensity AML therapies. Furthermore, following the precedent of *IDH1* mutations which are now considered favorable after the introduction of specific inhibitors, other gene mutations may be reclassified as new target therapies become available.

## 5. The Role of NGS in MRD Measurement and Monitoring

Measurable residual disease (MRD) has become increasingly important in AML patients for guiding therapy—such as selecting optimal consolidation strategies, maintenance therapy, follow-up, or donor lymphocyte infusions (DLI), for monitoring relapse, and as a prognostic biomarker [123,124].

The 2021 update on MRD in AML, from the European LeukemiaNet MRD working party, recognizes NGS-based MRD monitoring as useful for refining prognosis in addition to multiparameter flow cytometry (MFC). However, current evidence is insufficient to recommend NGS-MRD as a stand-alone technique [125]. The main pitfall is the relatively low sensitivity of NGS methods for detecting very small MRD clones. At diagnosis, variants are considered significant if VAF > 5%; whereas NGS-MRD positivity, measured on genomic DNA, is provisionally defined as ≥0.1% VAF. Although NGS-MRD test negativity is defined as VAF < 0.1%, even results below this threshold may still be associated with adverse outcomes [125].

The advantage of NGS-MRD lies in its ability to detect clonal heterogeneity and track the gain or loss of mutations that may precede AML relapse. For example, *NPM1* mutations, generally considered stable markers for qRT-PCR monitoring [126], can become undetectable at relapse in about 10% of patients [127,128]. Therefore, analyzing a broader panel of genes allows better monitoring of clonal evolution in AML and the identification of emerging mutations predicting imminent morphologic relapse. Nevertheless, NGS-MRD has limitations, including low sensitivity without error-correction methods [129,130], and the detection of mutations—particularly in *DNMT3A*, *TET2*, and *ASXL1* (DTA genes)—that can occur in healthy individuals due to age-related clonal hematopoiesis. Germline mutations (typically showing VAF around 50%) are noninformative for MRD [125]. Additional limitations include genetic clonal heterogeneity and evolution over time [131,132,133], as well as the emergence or selection of subclones at relapse [134]. In a post hoc analysis of the MORPHO trial, MRD levels measured with NGS correlated remarkably with relapse-free survival (RFS) and the risk of relapse; moreover, MRD positivity at any level negatively affected RFS [135].

Despite the potential of NGS-based MRD assessment, it remains an investigational and non-standardized technique, and even the optimal time points for testing are still controversial and differ between laboratories [136]. However, two notable exceptions—*FLT3* MRD and a five gene NGS panel—are approaching integration into clinical practice.

The gene encoding fms-like tyrosine kinase-3 (FLT3) is one of the most frequently mutated genes in AML, being altered in approximately 25% of cases. FLT3 mutations most commonly consist of internal tandem duplications (ITDs), which can vary in length from as little as three base pairs (bp) to over 400 bp, while generally remaining in-frame and therefore resulting in a functional protein [137]. Each *FLT3*-mutated patient has a unique ITD sequence, making its detection and longitudinal monitoring with PCR-based MRD assays technically challenging. A noteworthy advanced laboratory technique has been developed by Invivoscribe^®^ and approved by the FDA. This method combines an initial PCR designed to amplify *FLT3*-ITD mutations, followed by NGS. Barcoded primers are used to target the area surrounding the juxtamembrane (JM) domain of the *FLT3* gene where ITDs typically occur, to selectively amplify the sequences of interest. The resulting PCR products are then purified and prepared for NGS analysis, enabling sensitive detection and quantification of ITD variants [138]. This assay is capable of detecting ITDs ranging from 9 bp to 252 bp and insertions from 3 bp up to the maximum detectable ITD size, with an analytical sensitivity of 5 × 10^−5^ mutant alleles per total alleles. A compelling demonstration of the clinical utility of this combined PCR/NGS method for the detection of *FLT3* MRD assessment was provided by the MORPHO trial. In this study, patients received gilteritinib for 2 years following allo-HSCT. Approximately half of the participants had measurable MRD either before or after transplantation., In a prespecified subgroup analysis, gilteritinib significantly improved outcomes in MRD-positive patients (HR, 0.515 [95% CI, 0.316 to 0.838]; *p* = 0.0065), but not in patients with undetectable MRD [139]. It is anticipated that this NGS/PCR approach for *FLT3* detection will likely be incorporated into future MRD testing guidelines [123].

The last example of NGS-based MRD is the pre-MEASURE study, which evaluated the prognostic value of a five-gene panel in patients with AML. Targeted error-corrected DNA sequencing was performed on pretransplant blood samples to detect variants in *FLT3*, *NPM1*, *IDH1*, *IDH2*, and *KIT*. The persistence of FLT3-ITDor *NPM1* variants in the blood at an allele fraction of 0.01% or higher was associated with increased relapse rates and worse survival compared with patients lacking these variants [140]. Moreover, in the ALFA0702 study (NCT00932412) an error-corrected NGS-based MRD assessment demonstrated that the persistence of non-DTA mutations (HR = 2.23 for RFS and 2.26 for OS), and DTA mutations (HR = 2.16 for OS) was associated with poorer prognosis in multivariate analysis [141].

## 6. The Role of NGS in the Detection of Germline Predisposition

When approaching a newly diagnosed AML patient, we now have an unprecedented opportunity to detect germline predisposition simply by performing a standard diagnostic NGS panel. In a Korean cohort of AML patients, the prevalence of germline mutations was 7.2% [142,143]. A mutation may be suspected to be of germline rather than acquired somatically when the VAF is relatively high, typically ≥30–40% [144,145]. Therefore, NGS has revolutionized the traditional clinical concept of “familial MDS/AML”, previously defined by the occurrence of acute leukemia, myeloid malignancies, or characteristic cytopenias in at least two or more first- and/or second-degree relatives in the same family with at least one case being diagnosed as MDS or AML [146]. Conversely, when performing diagnostic NGS, clinicians should be aware, and ideally inform patients in advance, that testing at the time of diagnosis of AML/MDS diagnosis may uncover germline mutations, potentially reveal familial predisposition. Such findings may bring significant clinical, psychological, and social implications for patients and their relatives [146,147]. Moreover, the presence of germline mutations conferring familiar predispositions can have an impact on the choice of donor source when considering allo-HSCT from a relative. Although donor-derived leukemia is a rare event (about 1%), case reports of MDS arising from donor cells have been described [15,148,149,150]. The ELN-2022 guidelines outline clinical features that should prompt testing of germline predisposition, including at least two cancers, AML/MDS diagnosed at an unusually young age, a personal history of hematologic malignancy accompanied by a first- or second-degree relative within two generations with a hematologic cancer or a solid tumor before age 50, or the presence of other hematopoietic abnormalities [3]. Conversely, the Nordic MDS study group (NMDSG) proposed slightly different criteria for identifying patients who should be evaluated for myeloid neoplasms with germline predisposition. They also recommend confirming any pathogenetic variants detected by diagnostic NGS on a different tissue such as fibroblasts or peripheral blood collected during remission [145,151].

The 5th edition of WHO classification of hematolymphoid tumors categorized myeloid neoplasms with germline predisposition into three subgroups: myeloid neoplasms with germline predisposition without a preexisting platelet disorder or organ dysfunction (*DDX41*, *TP53*, *CEBPA*); myeloid neoplasms with germline predisposition and pre-existing platelet disorder (*RUNX1*, *ANKRD26*, *ETV6*); and myeloid neoplasms with germline predisposition and potential organ dysfunction (*GATA2*, Shwachman-Diamond syndrome (SDS) [152], Fanconi anemia (FA) [153], Down syndrome [154], etc.) [2,155].

### 6.1. DDX41

The most frequent genetic alteration associated with germline myeloid neoplasms is *DDX41*, accounting for about 80% of cases [156]. DEAD-box helicase 41 (*DDX41*), localized on chromosome 5q35, plays an important role in innate immune sensing and hematopoietic homeostasis. *DDX41* recognizes foreign or self-derived nucleic acids generated during microbial infection, thereby initiating an anti-pathogen response [157]. In one of the largest international studies, involving 346 patients with *DDX41* among 9082 patients with myeloid neoplasms (MN), *DDX41* accounted for 80% of germline predisposition to MN. Notably, in the Japanese male population, the incidence of *DDX41* mutations was 10 times higher than in than the general population. Furthermore, individuals with mutated *DDX41* have a lower risk of developing AML/MDS until around 40 years of age, but it increased to approximately 50% by 90 years of age [158]. Moreover, *DDX41* mutations appear to be associated with favorable prognosis when AML is treated with HMA plus venetoclax according to ELN2024 [121]; however, this observation is based on a limited number of patients [159,160], making these findings preliminary. Similarly, in patients treated with intensive chemotherapy, the presence of mutated *DDX41* in MDS/AML was reported to confer a favorable prognosis with longer OS compared to *DDX41* wild-type AML and other favorable-risk AML cases [161,162,163]. The protective effect of *DDX41* appears to mitigate the negative impact of other adverse markers like *TP53* [158]. Recommendations from the Nordic working group on germline predisposition for myeloid neoplasms include guidance for the surveillance of symptomatic carriers of germline *DDX41* variants [156].

### 6.2. TP53

The *TP53* gene encodes a protein considered one of the cell’s most crucial transcription factors, often referred to as the “guardian of the genome.” Due to its key functions, *TP53* is the most frequently mutated tumor suppressor gene in human cancers. Li-Fraumeni syndrome is caused by a pathogenic germline mutation in *TP53* and is linked to a markedly increased risk of various solid tumors—such as those of the breast, pancreas, central nervous system, and sarcomas—as well as blood malignancies like MDS, AML, and ALL. By the age of 70, the likelihood of developing cancer in affected individuals reaches nearly 100%. In these patients, myeloid hematologic malignancies often arise secondary to prior radiation or chemotherapy treatments administered for other cancers. Mutations in *TP53* greatly increase the risk associated with these blood cancers and are consistently correlated with a poor prognosis [164].

### 6.3. GATA2

The GATA-binding protein 2 (*GATA2*) plays a central role in blood stem cell generation and maintenance and directly modulates p53-induced apoptosis via the regulation of the MDM2 modulator RASSF4, which may contribute to chemo-resistance [165]. The GATA2 syndrome was first described in 2011 in patients presenting with monocytopenia, B cells, B-cell precursors, and NK cells deficiency, and/or absence of plasmacytoid dendritic cells in blood; late onset infections (non-tuberculous mycobacterial, fungal, or human papillomavirus (HPV) infections); pulmonary alveolar proteinosis; increased risk of progress to MDS or AML; and an autosomal dominant inheritance pattern due to haplo-insufficient mutations in one allele of *GATA2* [166]. Germline *GATA2* mutations account for approximately 15% of advanced and 7% of all primary MDS cases in pediatric population, making *GATA2* the most common pediatric germline mutation [167]. Although data on the efficacy of allo-HCST are limited to small cohorts of patients with somatic *GATA2*-mutated AML, transplantation appears beneficial, also reducing the risk of opportunistic infections [168]. Given that almost 200 likely unique pathogenic variants have been described, which can be classified into four groups: (1) truncating mutations (splice-site, nonsense, frameshift, and whole-gene deletions) proximal to or within the ZF2 domain; (2) missense mutations within the ZF2 domain; (3) mutations resulting in aberrant mRNA splicing (e.g., synonymous changes); and (4) other regulatory variants [169], it is essential to perform an NGS study of the entire gene to detect all possible pathogenetic variants.

### 6.4. RUNX1

The *RUNX1* gene encoding a key hematopoietic transcription factor was first identified in AML cases carrying translocation t(8, 21). It was initially named *AML1*. and later renamed *RUNX1* due to its homology to the *Drosophila* transcription factor *runt*, forming the “Runx” family of transcription factors [170]. In 1999 *RUNX1* germline mutations were linked to a familial platelet disorder with associated myeloid malignancy, a rare autosomal dominant disease characterized by thrombocytopenia and a predisposition to myeloid neoplasms. In a study of 27 families with *RUNX1* variants, half of the affected individuals had CHIP, and sequential sequencing data from 19 patients demonstrated dynamic changes in somatic mutations over time [171]. AML patients with *RUNX1* mutations have a dismal prognosis [172]. Since multiple alterations in *RUNX1* have been described, including multiexon or whole gene deletions, frameshifts, missense, and nonsense mutations [173], NGS is essential for their comprehensive detection. Furthermore, differential diagnosis with other familial disorders associated with thrombocytopenia requires sequencing of *ETV6* [174,175] and *ANKRD26* [176,177,178].

### 6.5. ETV6

The *ETV6* gene produces a transcriptional repressor belonging to the ETS family, playing a key role red blood cells and megakaryocytes maturation [175]. Germline *ETV6* mutations are inherited in an autosomal dominant manner and can lead to thrombocytopenia type 5, with risk—approximately 30%—of developing blood cancers. These malignancies can be either lymphoid (such as acute lymphoblastic leukemia, ALL) or myeloid (including AML). Similarly to other hereditary blood disorders, the onset of cancer usually requires acquisition of at least one additional somatic mutation in genes commonly implicated in leukemia development.

## 7. The Impact of NGS in the Detection of Druggable Mutations at Relapse

In patients with AML relapsed after several lines of therapy or after allogeneic transplantation, the prognosis is extremely poor, making it necessary to activate early palliative care or to enroll patients in clinical trials whenever feasible [179,180,181]. However, in the near future, NGS may become a compass to guide targeting actionable genes detected in r/r AML patients. An Italian case report of two patients demonstrated clonal and sub-clonal evolution during disease progression highlighting how NGS can help to uncover new therapeutic targets [182]. In a more robust dataset of 1470 AML patients, NGS was used to analyze 17 potentially actionable genes (*ALK*, *CSF1R*, *FGFR1/2/3*, *FLT3*, *IDH1/2*, *JAK2*, *KDR*, *KRAS/NRAS*, *NPM1*, *PDGFRA*, *PTPN11*, *RET*, and *TP53*), because these genes are directly or indirectly targetable with standard (with 6% of patients receiving off-label agents) or investigational agents (53% of these patients were enrolled in clinical trials) [183]. The main pitfall of this approach is that many mutations identified by NGS are either linked to drugs not yet approved, or cannot currently be targeted with available treatments (e.g., *TP53*). Nevertheless, NGS promises to improve survival and quality of life of older adults [184].

## 8. Discussion

The advent of faster and less expensive NGS techniques promises to revolutionize the field of MN. There are several gene panels available (e.g., the Illumina TruSight Myeloid panel, the Archer VariantPlex Core Myeloid panel, the Human Myeloid Neoplasms QIASeq DNA Panel, and the AmpliSeq for Illumina Myeloid panel). The presence of several translocations can be detected using a single diagnostic test based on RNA-based NGS (RNA sequencing), reducing costs and preserving laboratory resources from extensive cytogenetic analyses, and multiple specific real-time quantitative PCR for selected transcripts [185]. With RNA sequencing of the known gene partner, finding other rare partners has become easier (e.g., *KMT2A*) [186]. Moreover, the possibility to sequence long genes, that present technical difficulty to sequence (e.g., *CEBPA* is a GC-rich gene which is particularly difficult to amplify by PCR or *TP53*), can help clinicians to assign a patient to a more appropriate risk group. Nevertheless, we still have a limited number of drugs in the first-line setting, reducing the possibility to offer each patient a personalized approach and precision medicine. In a retrospective study, patients were reevaluated in light of newly available NGS and cytogenetic data; however, no statistically significant difference in survival was found between the two groups [100].

For patients with MDS or AML, *TP53* mutations probably represent one of the most negative prognostic markers, given their association with low sensitivity to chemotherapy (including venetoclax-based treatments) and substantially worse responses after HSCT [187,188,189,190]. Therefore, sequencing *TP53* is of utmost importance, despite the technical challenges that remain, such as the presence of VUS being not yet fully covered by current databases [191]. Unfortunately, no p53-based therapeutics have been approved to date, probably because, as a nuclear transcription factor, p53 does not possess typical drug target features and has therefore long been considered undruggable [192]. Another hot topic is the use of NGS as an MRD marker, which can help to refine the selection of AML patients for transplant in first complete remission and to guide the decision on post-transplant maintenance therapy (e.g., gilteritinib in MRD-positive patients) [139]. Moreover, patients who relapse often exhibit clonal evolution and biological shifts that complicate treatment, but NGS offers an opportunity to study the specific mechanisms of resistance to targeted drugs such as FLT3-inhibitors [193,194]. In the future, personalized medicine could be applied to patients rapidly identified by NGS; for example, Menin inhibitors for KMT2A-rearranged AML [93].

## 9. Conclusions

Reading the final report of an NGS exam in a patient with AML or MDS is a challenging task. Clinicians should be aware of both the potential and the limitations of the techniques employed by their laboratory, including the platform used, sequencing depth, and the interpretation of identified variants. VAF is another critical parameter that should be considered especially in patient harboring mutations typical of clonal hemopoiesis (e.g., *DNMT3A*, *SRSF2*, *TET2*), as it reflects the dimension of the clone at diagnosis and during follow-up. Another important concept is that not all mutations in the same gene are equivalent, and some cases still have unknown clinical significance (VUS). These variants require consultation of specialized databases, which may provide limited information that can change with time since some VUS can be reclassified when new evidence becomes available [195]. We recognize that currently clinicians are navigating the growing complexity of molecular data in daily practice, therefore we encourage direct interaction with the biotechnologists and bioinformatics involved in NGS analysis and report preparation. Moreover, for patients with suspected germline predisposition to MN a genetic counseling and multigene panel testing are recommended [196]. In conclusion, the dialog between hematologists and biotechnologists about the NGS report should not be intended as the endpoint, but rather as the beginning of a multidisciplinary discussion, particularly in the most challenging cases.

## 10. Future Directions

The field of NGS in AML and MDS is changing fast, with increasing availability of faster, simpler, and more affordable sequencing platforms. Clinicians are gaining experience and confidence in interpreting NGS reports of their patients. We believe this will profoundly influence our clinical practice, because NGS will likely be performed for each patient, including elderly ones. The complexity of the field may appear overwhelming at first glance and include the need to consult website-based databases of pathologic variants. Nevertheless, the integration of algorithms of Artificial Intelligence, could simplify the task of the hematologist and help avoid analysis paralysis [197,198].

## Figures and Tables

**Table 1:** This table shows the main genes mutated in AML or MDS, organized by functional category, with brief mechanisms of their alteration.

Functional Category	Example Genes/Alterations	Consequences of Genetic Alteration
Signal Transduction	*FLT3*, *NRAS*, *KRAS*, *c-KIT*, *PTPN11*	Confers proliferative advantage through the hyperactivation of signaling pathways such as JAK/STAT, PI3K/AKT, and RAF/MEK/ERK.
Myeloid Transcription Factors	*RUNX1*, *CEBPA*, or fusions like *RUNX1::RUNX1T1*, *PML::RARA*, *CBFB::MYH11*	Causes transcriptional deregulation, leading to impaired or blocked normal hematopoietic differentiation.
Tumor Suppressor Genes	*TP53*, *WT1*, *PHF6*	Deregulates normal transcription and disrupts cell cycle checkpoints and responses to cellular stress, often resulting in increased proliferation and impaired differentiation.
Spliceosome Complex	*SRSF2*, *SF3B1*, *U2AF1*, *ZRSR2*	Alters the proper maturation of mRNA, causing events such as intron retention or exon skipping, which can result in dysfunctional proteins.
Multifunctional Protein	*NPM1*	Nucleophosmin mutations cause abnormal cytoplasmic localization of the protein, disrupting ribosome biogenesis and the stability of tumor suppressors such asp53.
Cohesin Complex	*SMC1A*, *SMC3*, *STAG2*, *RAD21*	Affects chromosomal segregation and gene expression by altering chromatin accessibility, resulting in increased proliferation and impaired differentiation.
DNA Methylation	*DNMT3A*, *TET2*, *IDH1/2*	Leads to global changes in the epigenetic landscape by altering DNA methylation patterns, thereby affecting gene expression.
Chromatin Modifiers	*ASXL1*, *EZH2*, or fusions involving *KMT2A*	Perturbs epigenetic homeostasis through aberrant histone modifications, resulting in widespread changes in gene transcription.

**Table 2:** Recommended NGS tests for specific clinical questions in the diagnosis, management, and follow-up of MDS and AML.

Clinical Question	Recommended NGS Test	Rationale	Main Limitations
Initial diagnosis of MDS/AML	Targeted DNA NGS panel (20–50 genes)	Detect recurrent mutations relevant for WHO/ICC 2022 classification, ELN risk stratification, IPSS-M, and therapeutic targets.	Does not detect unknown fusions; reduced sensitivity for long ITDs, large indels, GC-rich regions
Suspected translocation or known gene fusion	Targeted RNA-seq for fusion detection	Identifies common/known fusions (KMT2A, RUNX1/RUNX1T1, CBFB/MYH11, NUP98, etc.).	Does not detect rare/novel fusions; requires high-quality RNA
Search for rare/unexpected gene fusions	Whole-transcriptome RNA-seq (WTS)	Detects novel/atypical fusions; provides gene expression and isoform profiling. Useful when cytogenetics is inconclusive.	Expensive, long turn-around time, requires advanced bioinformatics
Suspected germline predisposition	WES (tumor ± germline)	Broad analysis of coding genome; detects germline predisposition variants.	Uneven coverage; limited SV detection; moderate depth
Suspected complex structural variant (e.g., long FLT3-ITD, MLL-PTD, large indels, GC-rich amplicons)	Targeted long-read NGS	Ideal for complex alterations such as variable FLT3-ITD, MLL-PTD, CALR type 1, GC-rich CEBPA; resolves complex rearrangements.	Lower accuracy for SNVs; limited availability; higher costs
MRD monitoring (known variant)	Ultra-deep targeted NGS (DNA) or PCR-NGS	Very high sensitivity. Ideal for FLT3, IDH1/2, etc.	Requires an index variant; cannot identify new mutations
Confirmation and characterization of complex alterations	Whole-genome sequencing (WGS)	Covers the entire genome, including non-coding regions, SVs, CNVs, and cryptic translocations.	High cost; relatively low depth (30–60×); less sensitive for low VAF variants

## Data Availability

No new data were created or analyzed in this study.

## Abbreviations

The following abbreviations are used in this manuscript:

AML	Acute myeloid leukemia
AMP	Association for Molecular Pathology
CAP	College of American Pathologists
CCUS	Clonal cytopenia of undetermined significance
CHIP	Clonal Hematopoiesis of Indeterminate Potential
cnLOH	Copy-neutral loss of heterozygosity
CMML	Chronic myelomonocytic leukemia
HMA	Hypomethylating agents
HR	Hazard ratio
HSCT	Hematopoietic stem cell transplantation
ICC	International Consensus Conference
LOH	Loss of heterozygosity
MDS	Myelodysplastic neoplasms
MN	Myeloid neoplasms
MRD	Measurable residual disease
NGS	Next-generation sequencing
OS	Overall survival
RFS	Relapse free survival
SBS	Sequencing by synthesis
VAF	Variant allele frequencies
VUS	Variants of uncertain significance
WES	Whole-exome sequencing
WGS	Whole-genome sequencing
WHO	World Health Organization
